# Macrophage Activation Syndrome in Children: Update on Diagnosis and Treatment

**DOI:** 10.3390/children11070755

**Published:** 2024-06-21

**Authors:** Jin Lee, Kil Seong Bae, Jung Woo Rhim, Soo-Young Lee, Dae Chul Jeong, Jin Han Kang

**Affiliations:** 1 Department of Pediatrics, College of Medicine, The Catholic University of Korea, Seoul 06591, Republic of Korea; pedleejin@songeui.ac.kr (J.L.); cosmo7@catholic.ac.kr (K.S.B.); jwrhim@catholic.ac.kr (J.W.R.); dcjeong@catholic.ac.kr (D.C.J.); kjhan@catholic.ac.kr (J.H.K.); 2 Department of Pediatrics, Incheon St. Mary’s Hospital, The Catholic University of Korea, Incheon 21431, Republic of Korea; 3 Department of Pediatrics, Eunpyeong St. Mary’s Hospital, The Catholic University of Korea, Seoul 03312, Republic of Korea; 4 Department of Pediatrics, Daejeon St. Mary’s Hospital, The Catholic University of Korea, Daejeon 34943, Republic of Korea; 5 Department of Pediatrics, Bucheon St. Mary’s Hospital, The Catholic University of Korea, Bucheon 14647, Republic of Korea; 6 The Vaccine Bio Research Institute, College of Medicine, The Catholic University of Korea, Seoul 06591, Republic of Korea; 7 Department of Pediatrics, Seoul St. Mary’s Hospital, The Catholic University of Korea, Seoul 06591, Republic of Korea

**Keywords:** macrophage activation syndrome, children, diagnosis, treatment, review

## Abstract

Macrophage activation syndrome (MAS) is potentially fatal; so, early diagnosis and timely treatment are essential. However, detecting MAS is sometimes challenging because its principal features can be observed in other pediatric diseases that cause severe inflammation. Cytokine storm due to immune dysregulation represents the clinical and laboratory features of MAS that are included in the diagnostic criteria. Most cases of MAS occur as an underlying condition worsens and progresses. Therefore, a patient with autoimmune or autoinflammatory disease who shows unexplained clinical deterioration despite appropriate management should be considered at high risk for MAS (i.e., occult MAS). The basic principles of treatment are control of triggering factors, supportive care, and relief of hyperinflammation. Systemic steroids and cyclosporine A are frequently used as a first-line treatment. For the treatment of refractory MAS, cytokine-specific biologic agents such as anakinra have recently become preferred over traditional immunosuppressive agents such as etoposide. MAS might be underrecognized in pediatric patients with infectious and inflammatory diseases due to its diverse clinical presentations. Clinical suspicion of MAS is of the utmost importance for early recognition of the disease.

## 1. Introduction

Macrophage activation syndrome (MAS), part of the spectrum of hemophagocytic lymphohistiocytosis (HLH), is a hyperinflammatory phenomenon caused by uncontrolled activation of T cells and macrophages secondary to infections, malignancies, or rheumatic diseases [1,2]. This immunological catastrophe leads to massive hypersecretion of proinflammatory cytokines (i.e., cytokine storm), which manifests as persistent fever, splenomegaly, cytopenia, and multi-organ dysfunction [3]. Systemic juvenile idiopathic arthritis (sJIA) is the most common cause of MAS in children. MAS is also seen in other autoimmune or autoinflammatory diseases, including systemic lupus erythematosus (SLE), Kawasaki disease (KD), juvenile dermatomyositis (JDM), and periodic fever syndrome [4,5].

MAS is one of the most common pediatric rheumatic emergencies and is potentially fatal, with a mortality rate of 8–22% [6]. Therefore, early recognition and timely treatment of MAS are important for achieving a good prognosis [7]. However, detecting MAS in children can be difficult because the principal features of MAS, such as fever, thrombocytopenia, and liver dysfunction, are often observed in severe infections or flares of underlying rheumatic disease [7,8]. Understanding the characteristics and approaches of MAS is useful to avoid overlooking MAS in various systemic inflammatory diseases. The purpose of this review is to provide an overview of MAS in children and describe updates on diagnosis and treatment.

## 2. Overview

### 2.1. Classification and Terminology

MAS belongs to a group of disorders known as HLH. For educational purposes [9], HLH can be classified into two groups: primary (familial or genetic) and secondary (sporadic or acquired). Primary HLH is an autosomal-recessive genetic disorder that presents early in life, usually in infancy [10]. Secondary HLH can present at any age and develops as a complication of various medical conditions [11,12]: infection-associated (e.g., Epstein–Barr virus [EBV]), malignancy-associated (e.g., lymphoma), or rheumatic disease-associated (e.g., sJIA) (Table 1).

In 1985, Hadchouel et al. [13] reported a clinical syndrome of acute hemorrhagic, hepatic, and neurologic abnormalities in seven patients with sJIA. A few years later, the term MAS was used by the same group of investigators, who found evidence of activation of the monocyte–macrophage system in patients with sJIA and noticed that its clinical features were very similar to those observed in primary HLH [14]. In Korea, Kim et al. [15] reported a case of sJIA with splenomegaly, pancytopenia, hepatitis, and coagulopathy in 1988. Fortunately, the 9-year-old boy recovered with intensive care and high-dose steroid treatment. However, it took more than ten years for those authors to realize that the boy in their report was the first Korean patient with MAS.

Most early cases of MAS were reported in patients with rheumatic disease such as sJIA. Therefore, rheumatic disease-associated secondary HLH was previously called MAS. Because it has been reported in various medical conditions other than rheumatic disease, some experts use the term MAS interchangeably with secondary HLH [16,17]. Infection-associated secondary HLH is also called infection-associated hemophagocytic syndrome (IAHS) [18].

### 2.2. Epidemiology

MAS can complicate any inflammatory condition at onset or during the course of the disease [1,2,3]. In a study of sJIA [19], the median interval between sJIA diagnosis and MAS onset was 4 months, and the male to female ratio of patients was 4:6. In summarizing the epidemiologic studies on pediatric MAS, the approximate incidence would be listed in the following order: sJIA (~10%) > SLE (~5%) > KD (~2%) > JDM (18 cases reported worldwide) [20,21,22,23,24]. A similar incidence of MAS has been reported in an adult study [25]: adult-onset Still’s disease (AOSD, 11.5%), SLE (5.1%), systemic sclerosis (2.3%), dermatomyositis/polymyositis (DM/PM, 2.0%), and rheumatoid arthritis (RA, 1.5%). It would be prudent to remember that a considerable number of patients have an occult or subclinical form of MAS. Behrens et al. [20] reported that in sJIA, occult MAS was at least three times more prevalent than overt MAS.

Despite its clinical significance, the incidence of MAS can be underestimated in children with various infectious or inflammatory diseases [5,8]. As seen in the first Korean case of MAS [15], when a patient suddenly develops severe systemic inflammation and multi-organ dysfunction, most clinicians first consider the possibility of a serious bacterial infection such as sepsis, regardless of the type of underlying disease. However, not only infectious diseases but also autoimmune and autoinflammatory diseases can cause systemic inflammation and organ dysfunction. Pneumococcal infection plus systemic inflammation (i.e., systemic inflammatory response syndrome [SIRS]) is called pneumococcal sepsis, and pneumococcal sepsis plus multi-organ dysfunction is called pneumococcal multi-organ dysfunction syndrome (MODS) [26]. Similarly, sJIA with systemic inflammation is called severe sJIA (i.e., sJIA flare), and severe sJIA with multi-organ dysfunction can be called sJIA with MODS. The most well-known form of sJIA with MODS is sJIA complicated with MAS (sJIA/MAS) [27]. In other words, MAS is part of sJIA, SLE, and KD and is the most severe form in the spectrum of these diseases [28].

### 2.3. Pathogenesis

The cellular and molecular mechanisms of MAS are not fully understood. Decades of related research have identified several important findings [10,29,30,31]: (1) The immune response itself, not the underlying diseases or triggering factors, causes the disease pathology; (2) MAS is an immune activation, rather than autoimmunity involving self-antigens; (3) the cytolytic dysfunction of CD8+ T cells and natural killer (NK) cells, rather than macrophages, is a key event in the pathogenesis of MAS; and (4) interferon gamma (IFN-γ), produced by CD8+ T cells, is a key mediator of MAS development. In fact, sustained IFN-γ release promotes the activation of macrophages, resulting in an overproduction of cytokines such as interleukin (IL)-1β, IL-6, tumor necrosis factor (TNF)-α, IL-18, and IL-12 [29]. These findings support a specific cytokine blockade as a promising strategy for treating MAS in children [30,31].

**Figure 1:**
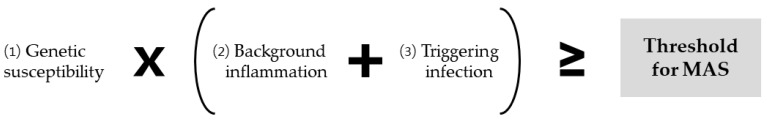
Threshold model [32,33]. MAS, macrophage activation syndrome.

MAS is not one disease but a heterogeneous group of diseases caused by different triggers and complex pathways. No single theory can explain the pathogenesis in detail, but the threshold model might be useful for understanding the basic principles of MAS development. Figure 1 shows the three components of the threshold model [32]: genetic susceptibility, background inflammation (i.e., underlying disease), and triggering infection. MAS occurs when the sum of those three component combinations exceeds the MAS threshold. In the pathogenesis of MAS, all three components contribute in varying proportions and ways. If there is a severe genetic defect in the granule pathway (i.e., primary HLH) or an abnormally strong triggering infection (e.g., *Leishmania*), the MAS threshold can be reached easily [31]. Theoretically, in the absence of genetic susceptibility, MAS does not occur even in the presence of a strong inflammatory background or triggering infection [33]. In addition, it is worth noting that there is some genetic overlap between MAS and primary HLH. For example, heterozygous mutations in primary HLH genes are observed in ~40% of sJIA patients who develop MAS [34,35].

### 2.4. Clinical and Laboratory Features

The principal features of MAS can be summarized in terms of systemic inflammation and organ dysfunction (Table 2). When MAS occurs, the clinical picture worsens more rapidly and more severely than seen during exacerbations of other pediatric infectious or inflammatory diseases [24]. MAS patients present with the so-called MAS triad of persistent fever, splenomegaly on abdominal examination, and cytopenia on blood tests. Thrombocytopenia is the earliest laboratory abnormality in MAS [16]. Increased levels of acute phase reactants such as C-reactive protein (CRP) and ferritin are also observed [19,36]. In practice, hyperferritinemia is the most prominent laboratory feature of MAS, often exceeding the upper limit of normal by 10–100 times [36]. Elevated liver aminotransferase levels may appear in the early stages of MAS and are often associated with disease activity [10]. Other laboratory features include hypertriglyceridemia, reduced NK cell activity, and increased levels of sCD25 (soluble IL-2 receptor [sIL-2R]; a marker of T cell activation), sCD163 (a marker of macrophage activation), and CXCL9 (an indicator of IFN-γ bioactivity) [37].

The extent of organ dysfunction observed in MAS patients is of great clinical im-portance because it directly affects the disease course and prognosis [38]. As seen in the first seven cases of MAS [13], hematologic (or hemorrhagic) and neurologic dysfunction are frequent. The hematologic problems manifest as epistaxis, petechiae, purpura, or ecchymoses [6]. Disseminated intravascular coagulation (DIC) with prolongation of prothrombin and partial thromboplastin times is also frequently identified in blood tests. As the disease progresses, fibrinogen is consumed, and the erythrocyte sedimentation rate (ESR) levels fall, which is one of the most distinctive laboratory features of MAS [39]. Hypofibrinogenemia and ESR drops might not be seen in the early stages of MAS or at all in some patients with MAS [40]. Approximately 20% of patients with MAS experience hematologic or hemorrhagic problems [39,40].

More than 30% of patients with MAS have neurologic abnormalities, including headache, seizures, ataxia, dysarthria, and altered mental status [41,42]. Encephalopathy, ab-normal electroencephalography, pleocytosis of cerebrospinal fluid (CSF), and posterior reversible encephalopathy syndrome (PRES) might also be seen [41]. Neurologic manifestations often herald a worse prognosis and should be monitored carefully [42]. In addition to hematologic and neurologic dysfunction, cardiopulmonary (e.g., shock or acute respiratory distress syndrome [ARDS]), gastrointestinal (e.g., abdominal pain or hematemesis), and renal (e.g., acute kidney injury [AKI]) involvement can be observed in patients with MAS [19,20].

## 3. Diagnosis

### 3.1. Diagnostic Criteria

Important clinical and laboratory features serve as the diagnostic criteria for MAS. Table 3 shows the three diagnostic tools that are commonly used in the field of pediatric MAS. The HLH-2004 diagnostic criteria were promulgated by the Histiocyte Society, and a diagnosis is made when a patient meets five or more of the eight criteria [43]. These criteria are diagnostically useful and have been the most widely applied to children with MAS during the past 20 years. However, they have some limitations in their application. They are sensitive for identifying primary HLH, but they are less sensitive for MAS because they were originally developed for primary HLH [4,8]. In addition, the NK cell activity and sCD25 assays included in the HLH-2004 criteria are not readily available to most clinicians [16]. To address those limitations, the Paediatric Rheumatology International Trials Organisation (PRINTO) proposed the 2016 classification criteria for MAS in patients with sJIA [44]. A diagnosis of MAS in sJIA requires hyperferritinemia (>684 ng/mL) plus any two of the following four criteria: platelet counts ≤ 181,000/μL, aspartate aminotransferase (AST) > 48 U/L, triglycerides (TG) > 156 mg/dL, and fibrinogen ≤ 360 mg/dL. The 2016 MAS classification criteria are also useful for diagnosing MAS in other autoimmune and inflammatory diseases such as KD and JDM [23,45].

Fardet et al. [46] proposed a new diagnostic tool for MAS or secondary HLH, called the hemophagocytic syndrome diagnostic score (HScore). Compared with the HLH-2004 criteria, the HScore includes immunosuppressive status and AST levels and excludes the NK cell activity and sCD25 assays. A summed HScore of greater than 169 has a sensitivity of 93% and specificity of 86% for MAS. Debaugnies et al. [47] reported that the HScore was more diagnostically appropriate for pediatric MAS than the HLH-2004 criteria. Recently, especially during the coronavirus disease 2019 (COVID-19) pandemic, the HScore has received clinical attention because it has frequently been used to diagnose COVID-19-associated cytokine storm syndrome in adults, COVID-19-associated multisystem inflammatory syndrome in children (MIS-C), and other COVID-19-associated hyperinflammatory conditions [48,49,50]. Each of these three tools has its own strengths and weaknesses; so, the method appropriate to the clinical situation should be applied. Because these diagnostic tools are complementary rather than competitive, some experts apply more than one method to correctly diagnose MAS [4,5].

### 3.2. Differential Diagnosis

Primary HLH is one of the most important diseases to distinguish from MAS. Typical primary HLH develops in infants younger than 1 year of age and presents with much more severe clinical manifestations than seen with MAS or other forms of HLH. Infants with primary HLH often show a critical sepsis-like appearance [11,12], and their clinical course worsens suddenly and unpredictably. Cakan et al. [51] suggested that genetic testing be performed in patients who have recurrent episodes or extremely high ferritin levels because they are more likely than others to have undiagnosed primary HLH.

In the pathogenesis of MAS, infections play an important role as triggering factors. However, severe infections such as sepsis and atypical infections due to leishmaniasis, tuberculosis, or adenovirus can cause diagnostic confusion with MAS [12]. For example, both MAS and severe or atypical infections often present with leukopenia or thrombocytopenia, high CRP or ferritin levels, and hemorrhagic or hepatic problems [52]. In other words, infection is not only a triggering factor but also a confounding factor. To distinguish MAS from severe or atypical infections, organomegaly (splenomegaly or hepatomegaly) and hypofibrinogenemia can be useful clues because they are common in MAS but rare in infections [39,40].

Some hematologic and oncologic diseases (Langerhans cell histiocytosis, Castleman disease, lymphoma, and unrecognized malignant diseases) can present MAS-like phenomena [3,12]. These diseases often present with leukocytosis or abnormal regional lymphadenopathy, which are not common in MAS. When a patient suspected of MAS has very high sCD25 levels but normal ferritin levels, further evaluation to exclude lymphoma is required [53]. On the other hand, normal sCD25 levels and very high ferritin levels indicate the possibility of an overlooked primary immunodeficiency (PID) disorder, especially in infants or young children [54]. Other important diseases on the differential diagnosis list for MAS include idiopathic acute liver failure, metabolic disorders, acute disseminated encephalomyelitis (ADEM), and autoimmune encephalitis [10,11,12].

### 3.3. Occult MAS: Who Needs Screening?

**Figure 2:**
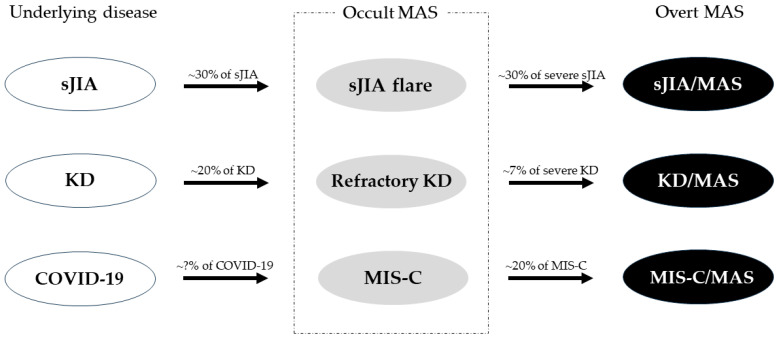
Occult MAS in actual practice [20,58,59]. MAS, macrophage activation syndrome; sJIA, systemic juvenile idiopathic arthritis; KD, Kawasaki disease; COVID-19, coronavirus disease 2019; MIS-C, multisystem inflammatory syndrome in children; sJIA/MAS, sJIA complicated with MAS; KD/MAS, KD complicated with MAS; MIS-C/MAS, MIS-C complicated with MAS.

The concept of occult MAS is useful for early recognition of MAS. Occult MAS is an inflammatory condition that does not currently meet the diagnostic criteria for MAS but might worsen and progress to overt MAS [20]. For example, when a patient with sJIA shows clinical exacerbation despite appropriate management (i.e., sJIA flare), they should be considered to have occult MAS [55]. Likewise, when a patient with KD shows a persistent fever despite intravenous immunoglobulin (IVIG) and aspirin treatment (i.e., refractory KD), they should be considered to have occult MAS [56]. In actual practice, a significant proportion of occult MAS (e.g., ~30% of sJIA flares and ~7% of refractory KD) progresses to overt MAS (Figure 2). In addition, occult forms of HLH or MAS may be more common than expected in infectious and malignant diseases [53,57].

Because MAS itself is a rare complication, not all children with systemic inflammatory diseases need to receive MAS screenings (i.e., tests for platelet count, liver transaminases, ferritin, LDH, triglycerides, fibrinogen, and D-dimer) [16]. However, MAS screening should be performed for subgroups at high risk of developing MAS (e.g., patients with sJIA flares, refractory KD, or MIS-C) [5].

### 3.4. Diagnostic Clues or Pitfalls

#### 3.4.1. Hemophagocytosis

Hemophagocytosis on a biopsy is an important histologic finding and strongly supports a diagnosis of HLH or MAS. However, the presence of hemophagocytosis is neither necessary nor sufficient for diagnosing HLH or MAS [16]. Minoia et al. [19] reported that evidence of hemophagocytosis was found in only 60% (150/247) of sJIA/MAS patients who underwent a bone marrow biopsy. In contrast, hemophagocytosis was observed in two-thirds of autopsies for critically ill patients with SIRS other than HLH or MAS [52,60]. That might be why hemophagocytosis was excluded from the 2016 MAS classification criteria. The diagnosis and treatment of MAS should not be delayed due to the presence or absence of hemophagocytosis [61].

#### 3.4.2. Hyperferritinemia

Hyperferritinemia, the most prominent laboratory feature of MAS, is included as a core criterion in all diagnostic tools for MAS. Although the cut-off ferritin levels are ≥500 ng/mL in the HLH-2004 criteria, most patients with MAS have ferritin levels > 1000 ng/mL, often >10,000 ng/mL [36]. Hyperferritinemia > 10,000 ng/mL is highly sensitive and specific for pediatric MAS and is often considered to be a pathognomonic finding of MAS [8,62]. Extreme hyperferritinemia (>100,000 ng/mL) might be associated with PID or primary HLH; so, a further evaluation for undiagnosed underlying disease should be performed [51,63]. A sharp increase in ferritin is seen at the onset of MAS, and there appears to be a good correlation between ferritin levels and inflammatory severity [6,24]. These findings indicate that repeated measurements of ferritin levels over time can be used to monitor disease activity, assess therapeutic response, and predict prognosis [10].

#### 3.4.3. Autoantibodies or Coronary Artery Abnormalities (CAAs)

The presence of autoantibodies or CAAs sometimes causes diagnostic confusion in patients with MAS. Just as hemophagocytosis is not specific for MAS, anti-nuclear antibodies (ANA) are not specific for SLE, and CAAs are not specific for KD [64,65]. In actual practice, sJIA/MAS or SLE complicated with MAS (SLE/MAS) can be mistaken for severe KD due to the presence of CAAs, and KD complicated with MAS (KD/MAS) can be mistaken for severe SLE due to the presence of ANA [66,67]. However, the presence of CAAs in MAS can indicate the severity rather than the cause of inflammation [60,65]. Likewise, the presence of ANA simply reflects the genetic background and does not appear to be associated with the likelihood of developing MAS [64]. Therefore, regardless of the presence or absence of so-called pathognomonic findings such as hemophagocytosis, ANA, or CAAs, MAS should be considered in children with unexplained fever, cytopenia, or organ dysfunction [5,38].

## 4. Treatment

### 4.1. Basic Principle

**Figure 3:**
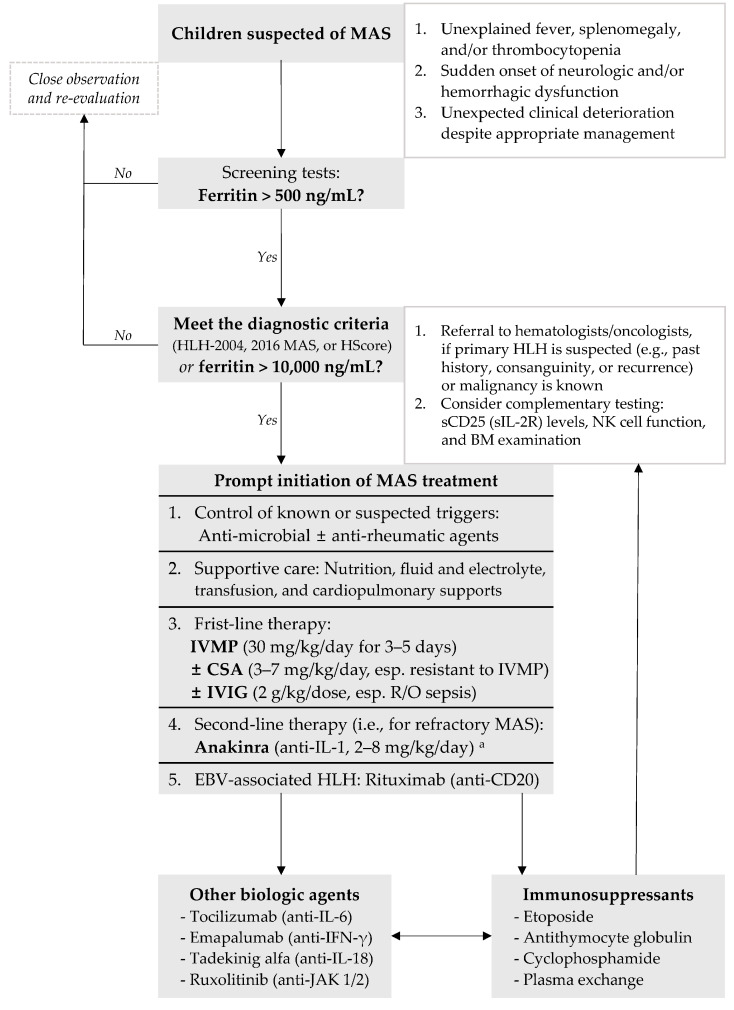
Therapeutic plan for treating MAS in children [10,24,71,72,73,74,75]. ^a^ Many institutions, especially in the United States, use anakinra instead of CSA as initial combination therapy with IVMP. MAS, macrophage activation syndrome; HLH, hemophagocytic lymphohistiocytosis; HLH-2004, HLH-2004 diagnostic criteria; 2016 MAS, 2016 classification criteria for MAS complicating sJIA; HScore, hemophagocytic syndrome diagnostic score; sIL-2R, soluble interleukin-2 receptor; NK, natural killer; BM, bone marrow; IVMP, intravenous methylprednisolone; CSA, cyclosporine A; IVIG, intravenous immunoglobulin; R/O, rule out; IL, interleukin; EBV, Epstein–Barr virus; IFN, interferon; JAK, Janus kinase.

Because no controlled trials have been conducted on MAS treatment, most MAS patients receive treatment based on expert opinion and previous case series [1,2]. The basic principles of treatment are controlling the known or suspected triggers, providing supportive care, and relieving hyperinflammation (Figure 3). In the management of sepsis, the first thing to do is find and control the focus of infection. Similarly, known or suspected triggers for MAS should be controlled using anti-microbial and/or anti-rheumatic agents unless contraindicated. Many MAS patients require admission to the intensive care unit (ICU) and support for organ dysfunction such as cardiopulmonary problems [24]. In addition, nutritional status and fluid and electrolyte balance should be closely monitored, and blood products might be needed to correct severe anemia and coagulopathy. To alleviate the hyperinflammation, non-specific immunosuppressants/modulators and cytokine-specific biologic agents are used.

### 4.2. First-Line Therapy

Systemic steroids (high-dose pulse intravenous methylprednisolone [IVMP] 30 mg/kg/day for 3 to 5 days) are most commonly used as first-line treatment [2,68]. Cyclosporine A (CSA) is a useful agent for initial combination therapy with IVMP or as adjunctive therapy for MAS resistant to IVMP [8,69]. Hematologists and oncologists might prefer etoposide over CSA as the initial combination therapy due to the HLH-2004 therapeutic protocol [43]. IVIG has also been used as an initial combination therapy with IVMP because the coexistence of MAS and infection is relatively common in children [6,40]. In a pediatric MAS study in India [70], 39% of patients received IVMP and CSA, 36% received IVMP alone, 19% received IVMP and IVIG, and 10% received IVMP, CSA, and IVIG as first-line therapy. If MAS does not respond to an initial combination therapy of IVMP, CSA, and/or IVIG, the condition is deemed to be refractory MAS, which requires second-line therapy.

### 4.3. Second-Line Therapy

The traditional immunosuppressants used to treat primary HLH and other forms of secondary HLH, such as etoposide or anti-thymocyte globulin, are still the adjunctive therapy of choice for refractory MAS in many institutions [2,10]. However, considering the potential complications of fatal myelosuppression and the opportunistic infections associated with those traditional therapies, biologic agents such as anakinra (anti-IL-1 agent) have recently been preferred [11,71]. Many studies indicate that refractory MAS shows an excellent response to anti-IL-1 treatment [31,72,73,74]. Although it might be less effective than anakinra, tocilizumab (anti-IL-6 agent) can also be used to treat refractory MAS, especially when anakinra is not available [74]. Rituximab (anti-CD20 agent) can be a helpful adjunct in the treatment of EBV-associated HLH [75]. Other cytokine-specific therapeutic options include emapalumab (anti-IFN-γ agent), tadekinig-alfa (anti-IL-18 agent), and ruxolitinib (anti-Janus kinase [JAK] 1/2 agent) [3,11]. Hematopoietic stem cell transplantation is a curative option for primary HLH, but it is not used in the routine management of pediatric MAS [2,24].

### 4.4. Treatment Monitoring and Duration

Appropriate monitoring of the therapeutic response in patients with MAS is as important as early recognition of the disease. To monitor disease activity, the leukocyte and platelet counts, aminotransferase and ferritin levels, and coagulation parameters can be serially checked [10,12]. Although they do not have readily available assays, sCD25 and sCD163 are also useful markers that correlate with disease activity [76].

Haytoglu et al. [77] found that most (83%) patients with MAS or secondary HLH successfully recovered after 8 weeks of treatment with simple immunomodulators instead of 40 weeks of complex chemotherapy. They suggested that patients who meet the diagnostic criteria for MAS are at risk of receiving overtreatment that is longer and more complex than necessary. According to the HLH-2004 protocol, patients with primary HLH can discontinue chemotherapy after the initial 8 weeks therapy if they have an adequate therapeutic response [43]. Similarly, patients with MAS should be closely monitored during their initial therapy to determine whether to continue or discontinue treatment [63]. After children recover from MAS, they need to be evaluated for complications of organ dysfunction and followed for recurrence of MAS. An unexpected clinical course might indicate the presence of an undiagnosed underlying condition, such as an immunodeficiency or rheumatic or malignant disease.

## 5. Special Consideration: MIS-C and MAS

Children with COVID-19 typically have milder clinical manifestations compared to adults [78]. However, some children develop severe COVID-19. In a study in North America (*n* = 2293) [79], approximately 30% of hospitalized children developed severe COVID-19, with a mortality rate of 0.5%. Severe COVID-19 causes ARDS, myocarditis, AKI, multi-organ dysfunction, MIS-C, COVID-19-associated hyperinflammatory syndrome (cHIS), and even HLH or MAS [49,50,79,80]. Table 4 shows the MIS-C case definition and proposed cHIS criteria, which are interesting in that they use the same parameters as the MAS diagnostic criteria.

It is clear that SARS-CoV-2 is a potential infectious trigger for HLH or MAS. Retamozo et al. [80] reported 28 adults and 30 children who developed HLH associated with SARS-CoV-2 infection. Like the pathogenesis of MAS, the development of severe COVID-19 appears to be related not only to the underlying disease but also to genetic susceptibility [49,50]. It would be clinically interesting to compare the principal features of MIS-C and MAS. MIS-C is a post-infectious complication of COVID-19 characterized by severe systemic inflammation and organ dysfunction [38,78]. Feldstein et al. [50] reported that all patients with MIS-C (*n* = 186) showed hyperinflammation and multi-organ (≥2) dysfunction, including gastrointestinal (92%), cardiopulmonary (80%), and hematological problems (76%). Many patients with MIS-C exhibit KD-like features that are included in the diagnostic criteria for KD, which sometimes leads to diagnostic confusion [83]. For example, MIS-C and KD/MAS are indistinguishable because both diseases share the same manifestations, including KD-like features, systemic inflammation, and organ dysfunction [84]. The most important difference between MIS-C and KD/MAS is whether SARS-CoV-2 has been identified as an infectious trigger [38,83].

Buda et al. [59] found that approximately 20% (59/274) of patients with MIS-C met the 2016 MAS classification criteria. Compared with MIS-C patients without MAS, MIS-C patients with MAS showed cytopenia and hypoalbuminemia more often, had higher levels of CRP and ferritin, and received more complex treatment. MIS-C can worsen and progress to MAS. Therefore, MIS-C should be considered a form of occult MAS in children (Figure 3). MIS-C and MAS have diagnostic overlap (such as hyperferritinemia) and therapeutic overlap (such as anakinra). Understanding the similarities and differences between MIS-C and MAS will provide useful clues for studying the pathogenesis of and therapeutic strategies for pediatric hyperinflammatory diseases [38].

## 6. Conclusions

Pediatric MAS is most frequently seen in sJIA patients and has been increasingly reported in SLE, KD, and JDM patients. The principal features of MAS, such as fever, thrombocytopenia, and liver dysfunction, can be observed in other systemic inflammatory diseases, leading to diagnostic confusion in actual practice. The HLH-2004 criteria, 2016 MAS classification criteria, and HScore are useful tools for diagnosing MAS in children. These tools are complementary rather than competitive; so, the appropriate methods depend on the clinical situation. Understanding the concept of occult MAS is useful for recognizing MAS early. For example, if a patient with sJIA or KD shows clinical exacerbation despite appropriate management, they should be considered to have occult MAS that might progress to overt MAS.

No therapeutic guidelines have been established for MAS; so, treatment is based on expert opinion and limited previous reports. First-line therapy consists of IVMP, CSA, and/or IVIG. For the treatment for refractory MAS, biologic agents such as anakinra have recently become preferred over immunosuppressants such as etoposide. Many patients with MAS achieve good outcomes from treatment with short-term immunomodulators instead of long-term complex chemotherapy. Therefore, the choice of treatment duration and modality should be determined based on individual therapeutic responses. During the COVID-19 pandemic, SARS-CoV-2 has become a potential trigger for HLH or MAS in children. Comparing the characteristics of pediatric hyperinflammatory diseases such as MIS-C and MAS will be an interesting future research topic.

MAS is a heterogeneous group of diseases with a broad spectrum. To avoid overlooking MAS in various systemic inflammatory diseases, MAS screening should be performed in children with unexplained fever, cytopenia, or organ dysfunction. Large-scale multicenter studies are needed to develop universally applicable diagnostic and therapeutic guidelines for MAS.

## Figures and Tables

**Table 1:** Classification of HLH [3,10,11,12].

	Susceptible Hosts	Principal Features	Related Conditions
Primary HLH	Infants with defects in the cytolytic pathway	Neurologic symptoms, fulminant disease course, consanguinity	*PRF1*, *UNC13D*, *STX11*, *STXBP2*, *RAB27A*, *LYST*, *AP3B1*
Secondary HLH			
Infection (i.e., IAHS)	Not determined	Clinical exacerbations despite proper anti-microbial treatments	EBV, CMV, HIV-1, HHV-6, mycoplasma, *Salmonella*, *Leishmania*, SARS-CoV-2
Malignancy	Adults with malignancy	Smoldering disease course	Lymphoma, leukemia, HCC, CAR-T
Rheumatic disease	sJIA and AOSD patients	Unexplained fever and cytopenia	sJIA, SLE, KD, JDM, AOSD, RA, DM/PM

HLH, hemophagocytic lymphohistiocytosis; IAHS, infection-associated hemophagocytic syndrome; EBV, Epstein–Barr virus; CMV, cytomegalovirus; HIV-1, human immunodeficiency virus 1; HHV-6, human herpes virus 6; SARS-CoV-2, severe acute respiratory syndrome coronavirus 2; HCC, hepatocellular carcinoma; CAR-T, chimeric antigen receptor T cell; sJIA, systemic juvenile idiopathic arthritis; AOSD, adult-onset Still’s disease; SLE, systemic lupus erythematosus; KD, Kawasaki disease; JDM, juvenile dermatomyositis; RA, rheumatoid arthritis; DM/PM, dermatomyositis/polymyositis.

**Table 2:** Clinical and laboratory/radiologic findings in MAS [6,19,20].

	Clinical Findings	Laboratory/Radiologic Findings
Systemic inflammation		
	Persistent fever	Increased levels of CRP, ferritin, TG, sCD25, sCD163, and CXCL9
	Splenomegaly and/or hepatomegaly	Hemophagocytosis in BM or LNs
		Decreased levels of ESR and albumin
Organ dysfunction		
Neurologic	Headache, seizures, ataxia, dysarthria, or altered mental status	Pleocytosis, abnormal EEG, or PRES
Hematologic/hemorrhagic	Coagulopathy	Anemia, leukopenia, thrombocytopenia
	Epistaxis, petechiae, purpura, or ecchymoses	DIC with prolongation of PT and PTT Hypofibrinogenemia
Gastrointestinal/hepatic	Abdominal pain or hematemesis	Elevated AST, ALT, LDH, and bilirubin
Cardiopulmonary	Shock or ARDS	ECHO or X-ray abnormalities
Renal	Edema, oliguria, or AKI	Elevated BUN and/or creatine

MAS, macrophage activation syndrome; CRP, C-reactive protein; TG, triglyceride; BM, bone marrow; LN, lymph node; ESR, erythrocyte sedimentation rate; EEG, electroencephalography; PRES, posterior reversible encephalopathy syndrome; DIC, disseminated intravascular coagulation; PT, prothrombin time; PTT, partial thromboplastin time; AST, aspartate aminotransferase; ALT, alanine aminotransferase; LDH, lactate dehydrogenase; ARDS, acute respiratory distress syndrome; ECHO, echocardiography; AKI, acute kidney injury; BUN, blood urea nitrogen.

**Table 3:** HLH-2004 criteria [43], 2016 MAS classification criteria [44], and HScore [46].

	HLH-2004	2016 MAS Classification	HScore (Points)
Fever	≥38.5 °C	(Fever ^a^)	38.4–39.4 °C (33); >39.4 °C (49)
Organomegaly	Splenomegaly	(Splenomegaly ^a^)	Hepato- or splenomegaly (23); both (38)
Abnormal CBC	Cytopenia ≥ 2 cell lines	Platelet counts ≤ 181,000/μL	2 cell lines (24); 3 cell lines (34)
Elevated liver enzymes	(Supportive evidence)	AST > 48 U/L	AST ≥ 30 U/L (19)
Abnormal TG	TG ≥ 265 mg/dL	TG > 156 mg/dL	133–354 mg/dL (44); >354 mg/dL (64)
or fibrinogen	or fibrinogen ≤ 150 mg/dL	Fibrinogen ≤ 360 mg/dL	≤250 mg/dL (30)
Hyperferritinemia	Ferritin ≥ 500 ng/mL	Ferritin > 684 ng/mL	2000–6000 ng/mL (35); >6000 ng/mL (50)
Elevated cytokines/APRs	sCD25 (sIL-2R) ≥ 2400 U/mL	–	–
Changes in NK cell activity	Low NK cell function	–	–
Hemophagocytosis	BM or LNs	–	Presence (35)
Infectious triggers	(e.g., IAHS)	(Frequent in many cases)	Known immunosuppression (18)
Organ dysfunction ^b^	–	–	–
Diagnosis	≥5/8 criteria	High ferritin + ≥ 2/4 criteria	Sum of points ≥ 169

^a^ Fever and splenomegaly are common in patients with sJIA. ^b^ Organ dysfunction is a principal feature of MAS but is not included in the MAS diagnostic tools. MAS, macrophage activation syndrome; HLH, hemophagocytic lymphohistiocytosis; HScore, hemophagocytic syndrome diagnostic score; CBC, complete blood cell count; AST, aspartate aminotransferase; TG, triglycerides; APRs, acute phase reactants; sIL-2R, soluble interleukin-2 receptor; NK, natural killer; BM, bone marrow; LN, lymph node; IAHS, infection-associated hemophagocytic syndrome.

**Table 4:** MIS-C definition [78,81] and proposed cHIS criteria [49].

	MIS-C Definition (CDC and RCPCH)	Proposed cHIS Criteria
Fever	>38.0 °C in children and adolescents	>38.0 °C
Organomegaly	Splenomegaly on USG	(Uncommon)
Abnormal CBC	Lymphopenia or thrombocytopenia	Anemia and thrombocytopenia ^a^
Elevated liver enzymes	Elevated AST, ALT, or LDH	AST ≥ 100 U/L or LDH ≥ 400 U/L
Abnormal TG	Elevated TG	TG ≥ 150 mg/dL ^b^
or fibrinogen	Abnormal fibrinogen	D-dimer ≥ 1.5 μg/mL
Hyperferritinemia	Elevated ferritin	Ferritin ≥ 700 ng/mL
Elevated cytokines/APRs	High IL-6, IL-10, or CRP (≥3 mg/dL)	IL-6 ≥ 15 pg/mL or CRP ≥ 15 mg/dL ^b^
Changes in NK cell activity	–	–
Hemophagocytosis	(Cases reported)	(Cases reported)
Infectious triggers	Evidence of SARS-CoV-2 infection	Evidence of SARS-CoV-2 infection
Organ dysfunction	Multi-organ: Gastrointestinal (92%), cardiopulmonary (80%), or hematologic (77%) [50]	(Frequent due to cytokine storm)
Diagnosis	High CRP + ≥ 2/5 organs + SARS-CoV-2 [82]	≥2/6 criteria (i.e., high risk)

^a^ Hemoglobin ≤ 9.2 g/dL and platelet counts ≤ 110,000/μL. ^b^ Cytokinaemia is defined as abnormalities in TG, IL-6, or CRP. MIS-C, multisystem inflammatory syndrome in children; cHIS, COVID-19-associated hyperinflammatory syndrome; CDC: Centers for Disease Control and Prevention; RCPCH: Royal College of Paediatrics and Child Health; USG, ultrasonography; CBC, complete blood cell count; AST, aspartate aminotransferase; ALT, alanine aminotransferase; LDH, lactate dehydrogenase; TG, triglycerides; APRs, acute phase reactants; IL, interleukin; CRP, C-reactive protein; NK, natural killer; SARS-CoV-2: severe acute respiratory syndrome coronavirus 2.
